# Sea urchin *Paracentrotus lividus* immune cells in culture: formulation of the appropriate harvesting and culture media and maintenance conditions

**DOI:** 10.1242/bio.039289

**Published:** 2019-02-04

**Authors:** Annalisa Pinsino, Andi Alijagic

**Affiliations:** Consiglio Nazionale delle Ricerche, Istituto di Biomedicina e Immunologia Molecolare ‘A. Monroy’, Via Ugo La Malfa 153, 90146 Palermo, Italy

**Keywords:** Echinoderm, Cell biology, *In vitro-ex vivo* model, Immunity, Marine invertebrate, Proxy to human

## Abstract

The sea urchin is an emergent model system for studying basic and translational immunology. Here we report a new method for the harvesting and maintenance of primary immune cells isolated from adult *Paracentrotus lividus*, a common Mediterranean sea urchin species. This optimised method uses coelomocyte culture medium, containing a high-affinity Ca^2+^ chelator, as the ideal harvesting and anti-clotting vehicle and short-term culture medium (≤48 h), and artificial seawater as the master medium that maintains cell survival and *in vitro-ex vivo* physiological homeostasis over 2 weeks. Gradually reducing the amount of anticoagulant solution in the medium and regularly replacing the medium led to improved culture viability. Access to a robust and straightforward *in vitro-ex vivo* system will expedite our understanding of deuterostome immunity as well as underscore the potential of sea urchin with respect to biomedicine and regulatory testing.

This article has an associated First Person interview with the first author of the paper.

## Abstract (precis)

**Summary:** Appropriate culture methods for sea urchin immune cells provide an invaluable and amenable model for answering immunological questions while limiting the use of mammalian organisms.

## INTRODUCTION

Sydney Ringer, an English physiologist in the early twentieth century, demonstrated for the first time that tissues and cells could be retained outside the body by culturing isolated animal hearts in a salt medium (Miller, 2004). Cell lines have revolutionised scientific research and are being used to test drug metabolism, cytotoxicity, antibody and vaccine production, the study of gene function, etc. (Kaur and Dufour, 2012). Despite being a powerful tool, immortalised invitromes – collections of cell lines that all relate to a single theme, such as the marine invitrome (Bols et al., 2017) – have the disadvantage of having altered or lost specific cell functions because of mutations. In contrast, primary cell culture represents much more accurately the biological microenvironment in which cells reside in tissues, as cell–cell signalling remains preserved; thus, primary cultures are a more appropriate tool for biotechnological applications and pathological investigations (Bols et al., 2017).

The principles reduce, refine and replace (3Rs) (Russell et al., 1959) have developed into imperative considerations in the design of scientific experiments that use animal models. Importantly, new and more sustainable methods, which minimise animal usage, have resulted in the development of novel *in vitro-ex vivo* methods specifically to address and limit the use of mammals. Only a few studies have addressed the development of marine invertebrate primary cultures (and these have focused on cells) derived from different tissues of a few species used for basic biological studies (response to pathogens, toxins, etc.) (Majeske et al., 2013; Vandepas et al., 2017; Maselli et al., 2018), even though primary cultures represent a rich source of cell and tissue types (Rinkevich, 2011). This limited understanding of marine primary cell cultures includes the absence of an appropriate medium formulation and a shortage of cell proliferation assessments (Cai and Zhang, 2014).

Sea urchins are marine deuterostome invertebrates and as Nobel legacy model organisms have been highly exploited for biological studies. In addition, the sea urchin *Paracentrotus lividus* has been nominated for inclusion on the list of alternative animal models presented by the EPAA (European Partnership for Alternative Approaches to Animal Testing). The full sequence release of the *Strongylocentrotus purpuratus* sea urchin genome (purple sea urchin) revealed the close genetic relationship between sea urchins and humans, an exceptional example of immune system complexity and sensing capacity (Sea Urchin Genome Sequencing Consortium, 2006), thus further reinforcing the relevance of this model organism. Immune cells function as the central sensing and effector components of the sea urchin *P. lividus* (phagocytes, amoebocytes and vibratile cells) reside within the coelomic cavity as well as in all other tissues, and orchestrate key innate immune functions, which consist of complement and cytokine secretion, chemotaxis, opsonisation, complement activation, phagocytosis and cytotoxic/cytolytic response. Immune cells produce and secrete specific regulatory biomolecules into the coelomic fluid (CF) (a ﬂuid with functions similar to the blood and the lymph of vertebrates), to maintain functional homeostasis and intercellular crosstalk (Smith et al., 2018; Pinsino and Matranga, 2015; Pinsino et al., 2015).

The establishment of suitable harvesting methods, a well-defined medium and long-term cultivation protocol, to result in a stable long-term *in vitro-ex vivo* system, is still needed. Previous sea urchin primary immune cultures, based on both simple and complex media, have not maintained satisfactory cellular viability over long periods (Johnson, 1969; Bertheussen and Seljelid, 1978; Dan-Sohkawa et al., 1993; Matranga et al., 2002; Matranga et al., 2006; Majeske et al., 2013). Here we formulated a physiological-like medium and developed successful methodologies to culture *P. lividus* immune cells, and have accomplished the following aims: (i) we developed a long-term, easy and reliable cultivation protocol and (ii) we compared different cell culture media already in use for sea urchin species for cell adherence, survival and growth. Our findings should further support the development of a new, powerful proxy for human immunology and toxicology studies.

## RESULTS AND DISCUSSION

### Quality control of freshly harvested sea urchin immune cells and related primary short-term cell cultures

**Fig. 1.**
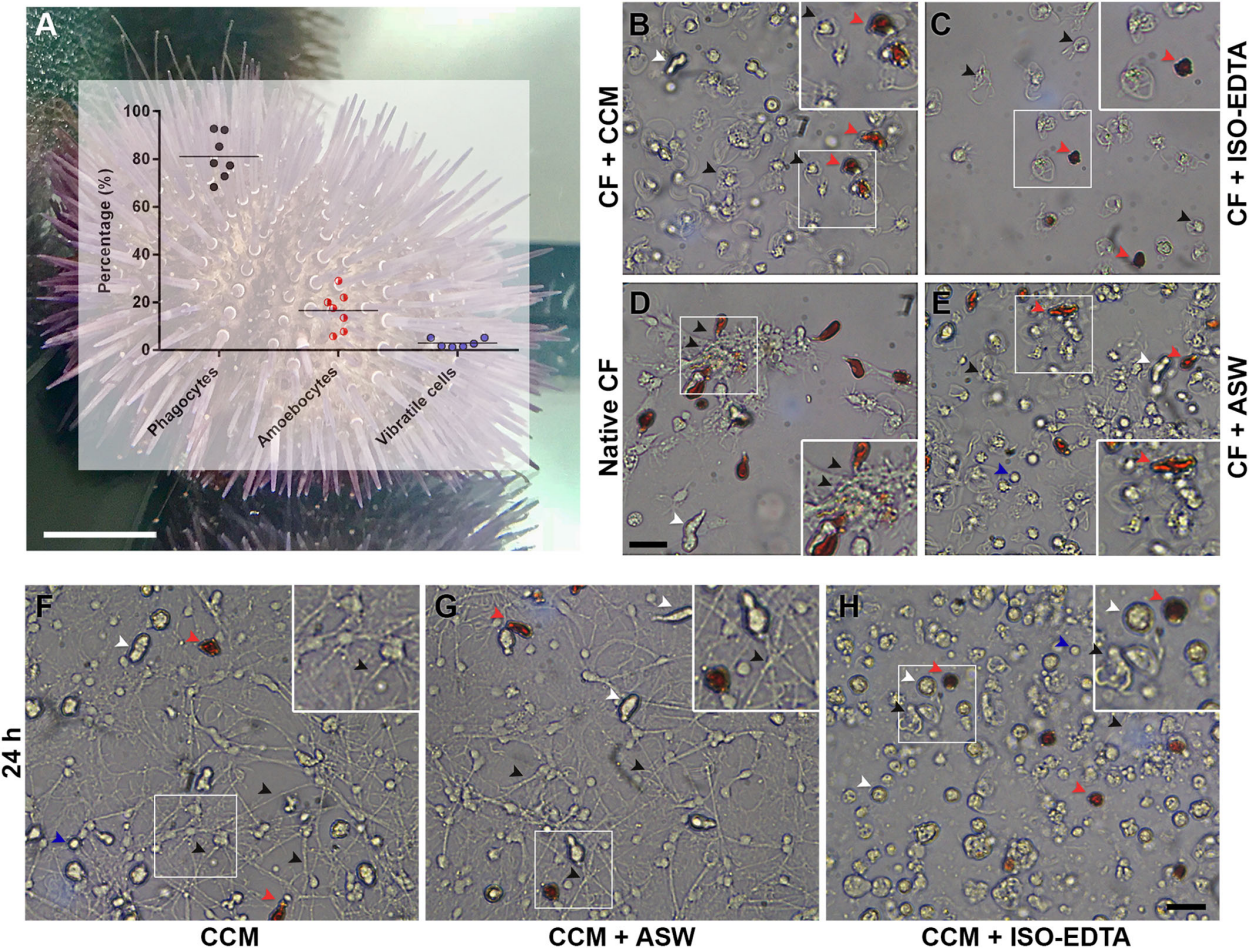
**Adult sea urchin and immune cells in culture under the microscope.** (A) Graphic representation of the three major immune cell types (phagocytes, amoebocytes and vibratile cells), in percent. A violet adult *P. lividus* maintained in an aquarium under controlled conditions. Scale bar: 1 cm. Immune cells were inspected under a microscope just after collection (B–E) and after 24 h in culture (F–H). Arrowheads with different colours indicate the major cell types of freely circulating *P. lividus* immune cells: red amoebocytes (red arrowheads), white/colourless amoebocytes (white arrowheads), phagocytes (black arrowheads), and vibratile cells (blue arrowheads). Live immune cells were collected in (B) CCM, (C) ISO-EDTA, (D) CF without an additional solution, and (E) ASW. (F) Immune cells cultured for 24 h in CCM show extensive spreading of the phagocytes and ovoid amoebocytes. (G) Immune cells collected in CCM and cultured for 24 h in CCM and ASW (1:1) supplemented with P/S display ovoid amoebocytes and long cytoplasmic processes of phagocytes that form an elaborated network. (H) Immune cells cultured for 24 h in ISO-EDTA show poor adhesion and morphological alterations. Scale bars: (B–H) 10 µm; white frames in B–H indicate inset images shown at a higher magnification.

The three major cell types of freely circulating immune cells have been described in *P. lividus* (phagocytes, amoebocytes and vibratile cells) (Pinsino and Matranga, 2015). Thus, a morphological analysis of the harvested cells in coelomocyte culture medium (CCM), ISO-EDTA and ASW as collected from sea urchins maintained under controlled conditions (Fig. 1A–E) was performed in a Fast-Read chamber under a microscope. Phagocytes are the most abundant immune cell type in all sea urchin species, and they have a dendritic-like phenotype that undergoes a striking morphological change as the result of a calcium-dependent clotting process that mediates the reorganisation of cytoskeletal microfilaments (Smith et al., 2018). The *P. lividus* phagocytes immediately collected in the anticoagulant solution containing EGTA (CCM) (Henson et al., 1999), were present in a petaloid form in which thin sheets of cytoplasm, like the petals of a flower, are organised around a central nuclear region (Fig. 1B, black arrowhead). Amoebocytes (red and white/colourless) appear ovoid, and vibratile cells are highly motile because of their long flagellum (Movie 1).

Phagocytes immediately collected in the anticoagulant solution containing EDTA (ISO-EDTA) (Matranga et al., 2000) were already undergoing the morphological transition from petaloid to frustrated filopodial shape with poor adhering capabilities (Fig. 1C). In contrast, amoebocytes are mainly rounded (Fig. 1C), and vibratile cells are not highly motile (not shown). EDTA-containing buffer did not preserve the morphological features of freshly collected cells, and this medium contained floating cells thus was not considered optimal for *P. lividus* immune cell culture. *Paracentrotus lividus* immune cells collected without anticoagulant aggregated under the well-known CF clotting process, in which phagocytes trigger the formation of the clot, thus aggregating single cells (filopodial phagocytes, amoebocytes, and probably vibratile cells) (Fig. 1D; Hillier and Vacquier, 2003). Interestingly, cells collected with ASW also tended to aggregate, albeit to a moderate extent, with phagocytes appearing petaloid and filopodial; amoebocytes remaining ovoid and vibratile cells, of which can be observed only the cell body, being highly dynamic (Fig. 1E, blue arrowhead). Both EDTA and EGTA are chelating agents, but whereas EDTA has the capacity to bind and sequester a variety of metal bivalent ions (Ca^2+^, Mg^2+^ and Fe^2+^), EGTA has a higher affinity for Ca^2+^. The genome of the sea urchin *S. purpuratus* revealed that a number of genes encode proteins most likely involved in cell–cell and cell–matrix adhesion (Whittaker et al., 2006). For example, toposome and amassin mediate the massive intercellular adhesion of sea urchin cells using calcium as a cofactor for adhesion (Hillier and Vacquier, 2003; Noll et al., 2007). Coagulation induced upon collection of CF in the absence of anticoagulant inhibited cell spreading and attachment to the bottom of the culture well. Our results highlight that CCM not only blocks the rapid clotting reaction and preserves the morphological features of freshly collected cells but also helps to maintain the suboptimal culture density and salinity very close that of the CF (Table S1), yielding a successful immune cell harvest. In fact, primary short-term cell cultures in CCM (24-h culture) indicated that this medium seems to maintain cells in a healthy state, as the adherent cells (phagocytes) were well dispersed over the bottom of the culture plate and were organised in bundles and fibres, and amoebocytes remained ovoid (83% of all initiated cultures were stable) (Fig. 1F; Movie 1). Immune cells cultured in CCM and ASW (1:1) showed morphologies similar to those of immune cells cultured only in CCM (Fig. 1G). In contrast, immune cells cultured in CCM and ISO-EDTA (1:1) were compromised and unstable, with many floating and rounded cells present in the culture difficult to recognise as a major known cell type (Fig. 1H).

### Real-time assays for determining immune cell viability, cytotoxicity and phagocytic ability in primary short-term cell cultures

**Fig. 2.**
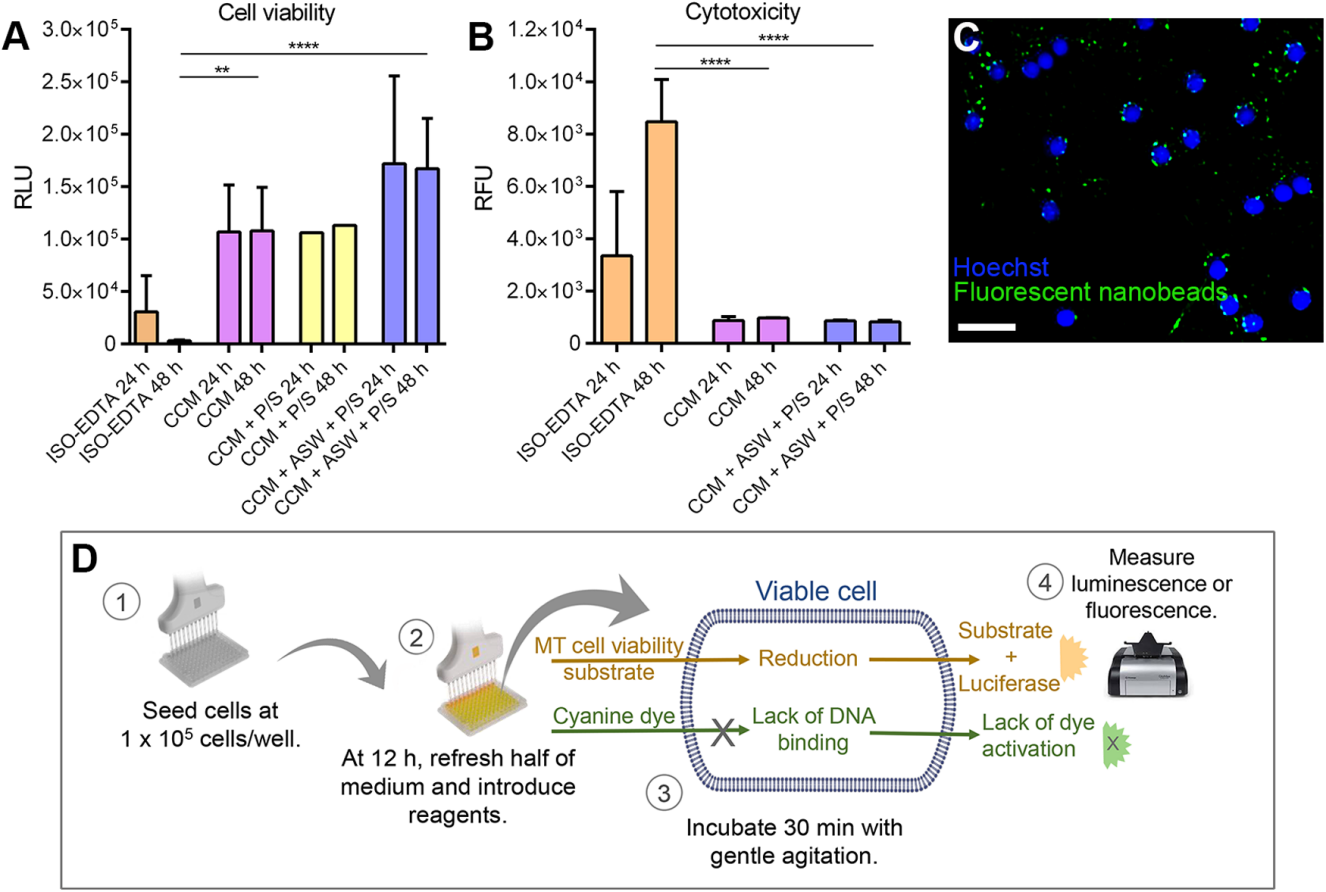
**Quantitative and qualitative validation of the short-term immune cell cultures by monitoring cell viability, cytotoxicity and phagocytic capability.** (A) Cell viability of cells cultured in ISO-EDTA, CCM, CCM with P/S and CCM+ASW with P/S for 24 and 48 h. (B) Cytotoxicity of the cells cultured in ISO-EDTA, CCM, and CCM+ASW+P/S. The data (A,B) represent the mean±s.d. from at least five biological replicates with one exception for cells cultured in CCM plus P/S. Stars indicate the significance of the differences between measurements: **P<0.01, ****P<0.0001. RLU, relative luminescence unit; RFU, relative fluorescence unit. (C) Immune cell nuclei labelled with Hoechst 33342 (blue colour); green channel indicates internalized fluorescent nanobeads. Scale bar: 10 µm. (D) A schematic representation of the cell viability and cytotoxicity procedures.

To support our morphological observation, after collection with CCM, the primary short-term cell cultures maintained in each medium (CCM, ISO-EDTA, ASW) with or without antibiotics were validated based on a continuous-read measurement of cell viability and cytotoxicity from 24–48 h (and intermediate time points, data not shown) by the GloMax Discover System. To the best of our knowledge, this is the first study in which such a system has been used for cell culture validation in echinoderms. Immune cells cultured in CCM (with or without antibiotics) exhibited high levels of viability (Fig. 2A–B). Culture efficiency was enhanced when after an initial 6 h period in culture, half of the medium was replaced with ASW, which removed non-adherent cells, reduced metabolic waste, promoted cell attachment and led to a maintenance of viability (with 82% of all initiated cultures resulting in a stable *in vitro* system) (compare Fig. 2 with Fig. 1G). In contrast, immune cells cultured in ISO-EDTA were significantly less viable. Phagocytosis, the primary innate immune response, remained intact *in vitro*, as determined by the encapsulation and internalisation of the fluorescent nanobeads (Fig. 2C, after 24 h culturing in CCM with ASW). Hoechst labelling of DNA in the CCM-ASW culture showed an even distribution of immune cells over the plate surface (Fig. 2C).

Antibiotics are routinely used in cell cultures to prevent bacterial infections; in our system, the aseptic conditions make these compounds not strictly necessary for short-term cultures. As an example, a host non-specific pathogen that causes bald sea urchin disease leads to cellular degranulation, phagocyte transitional arrest and decreased detection of cells carrying natural red pigment (echinochrome A) (Fig. S1). Echinochrome A is released by red cells as an iron-withholding strategy, which is one component of sea urchin innate immunity (Coates et al., 2018). Plating a sufficient number of cells per well significantly affected the likelihood of whether a culture would fail or succeed, whereas the presence or absence of antibiotics had no effect on this outcome. Diminished attachment capacity, a spherical morphology among amoebocytes and an absence of cell–cell interactions among immune cells were more likely to occur when a smaller number of cells were plated (Fig. S2).

### Long-term maintenance of *P. lividus* primary immune cell cultures

**Fig. 3.**
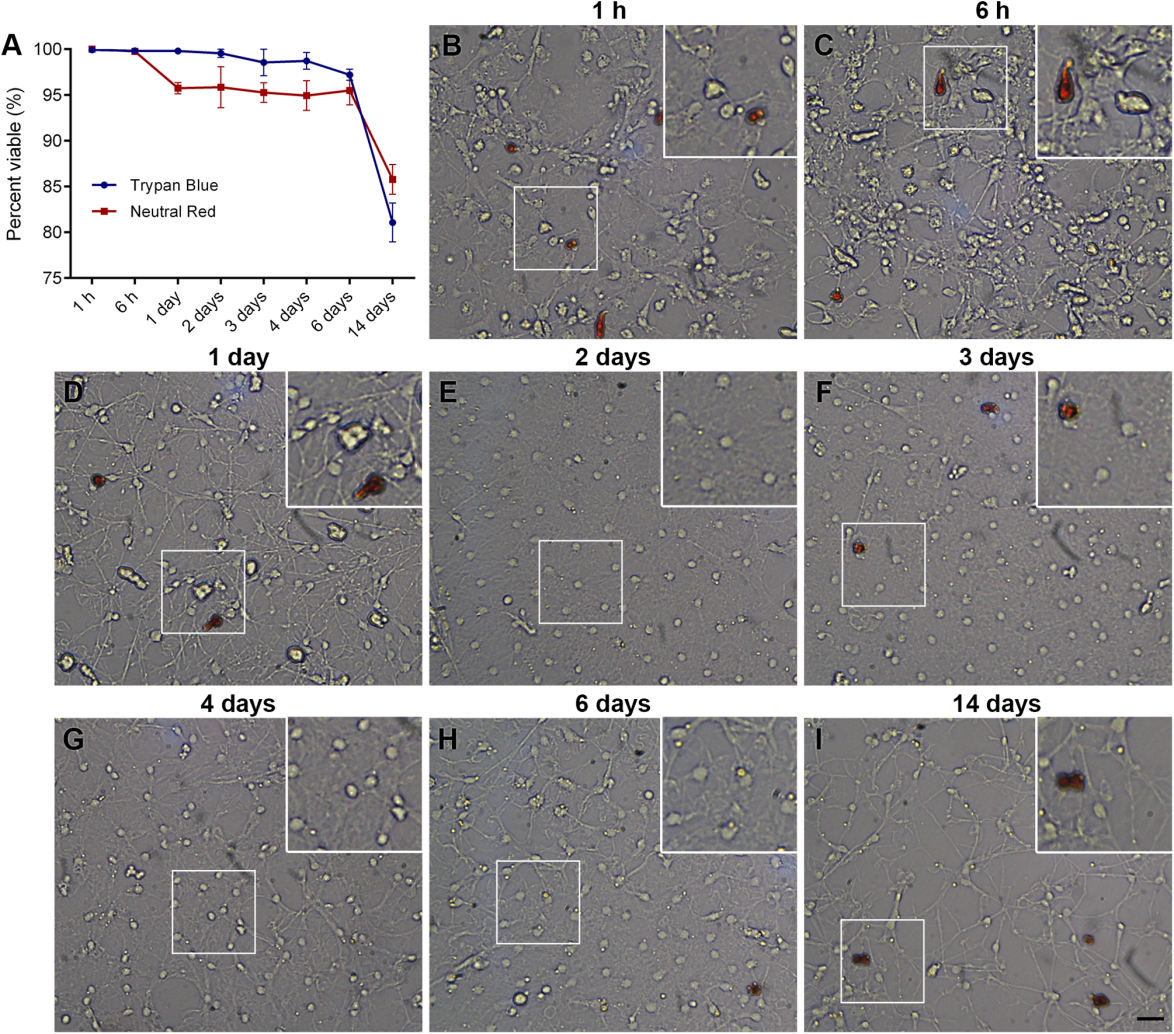
**Long-term maintenance of primary immune cell culture in CCM-ASW-based medium plus P/S.** (A) Graph showing the percentage of the Trypan Blue-negative cells and percentage of immune cells with the stable lysosomal membrane labelled with Neutral Red. The data represent mean±s.d. of at least three independent experiments. (B–I) Representative images of long-term immune cell cultures maintained with daily replacement of ASW. The culture show stable, well-adhered immune cells over the course of 2 weeks. Scale bar: 10 µm. White frames indicate the higher-magnification images shown in each inset. (B–I) Primary immune cells after (B) 1 h, (C) 6 h, (D) 1 day, (E) 2 days, (F) 3 days, (G) 4 days, (H) 6 days and (I) 14 days in culture.

Immune cells maintained in CCM-ASW-based medium exhibited low levels of cellular death and high levels of lysosomal stability and function for 2 weeks in culture (with more than 80% of stable cells in 14-day cultures) (Fig. 3A). *Paracentrotus lividus* primary immune cells, maintained in a monolayer of ‘adherent’ and ‘non-adherent cells’, followed a characteristic pattern composed of two phases: stationary and decline. Specifically, the viability of cultured immune cells was found to remain stationary during the first 6 days, and to decrease from 100% to 91% between 6 and 8 days, and from 90% to 83% between 9 and 14 days (data not shown). Cell viability slow decline could be due to apoptosis induced by the reduction in the number of the non-adherent cells (‘rare’ immune cells: vibratile cells and amoebocytes) after each medium change. A possible explanation for this result could be that the loss of these cells could change the expression of some signalling molecules sensing the environment on the adherent immune cell surface (Zhu et al., 2011), although other plausible explanations cannot be excluded.

Phagocytes immediately spread their dendritic protrusions across the surface of the plate and, when maintained in culture for longer periods, were able to form fibres while sustaining cell individuality (Fig. 3B–I). Amoebocytes morphology indicated that these cells were healthy, as they did not degranulate or shift toward a circular shape. As they are non-adherent cells, however, they were reduced in number after each medium change (Fig. 3B–I). In addition, after complete medium replacement, we did not observe the formation of new clotting structures (Fig. 3B–I). In contrast, numerous large multinucleated syncytia, without the clear cytoplasmic border between individual cells, were observed in the cases in which regular daily medium replenishment was stopped (Fig. S3). A possible explanation for this result could be an accumulation of the cellular particles and/or metabolic waste coming from the aggregation of the cells, a mechanism that seems to be considered as an immune trigger for clearance *in vitro* (Majeske et al., 2013). Critically, our findings support the idea that the *P. lividus* immune cell culture works as a simultaneously biphasic process; on the one hand, it is affected by a gradual decrease of anti-clotting molecules, and, on the other hand, it is affected by a new environment maintained by adhesive molecules that produce a new *in vitro-ex vivo* niche.

## CONCLUSIONS

The goals of our studies on sea urchin immune cell culture were to reduce the number of sea urchins used for experimentation, to minimise the need for *in vivo* studies and to refine the use of this model organism as a proxy to human. The sea urchin *P. lividus* is a common echinoid living in the northeast Atlantic ocean and the Mediterranean sea, but its populations have recently collapsed, and today it is an extremely rare species in some regions (Yeruham et al., 2015). Unfortunately, this species – which is an important food source for fishes and other animals, including humans – is facing increasing anthropogenic pressures in its coastal environments (Guidetti and Mori, 2005). It also has great potential as an experimental resource because many molecular and regulatory mechanisms of the immune response are conserved across many organisms, including humans (Alijagic and Pinsino, 2017). Thus, the methodologies presented here should facilitate the unravelling of complex cellular phenomena, and will be pivotal elements in establishing the sea urchin invitrome.

## MATERIALS AND METHODS

### Animal collection and maintenance

Specimens of sea urchin *P. lividus* (Lamarck, 1816) were collected from the sub-tidal rocky shores around the north and northwest coast of Sicily (Italy). Sea urchins were acclimatised and maintained under controlled conditions of temperature (16±2°C), pH (8.1±0.1), salinity (38–39‰) and density (1.028–1.030 g/cm^3^), depending on the season, in tanks supplied with flow-through oxygenated Artificial Seawater (ASW) (Aqua Ocean Reef Plus Marine Salt, Aquarium Line, Italy). Animals were fed every 7 days with the green alga *Ulva lactuca*.

### Animal handling and immune cell harvesting

Syringes (1 ml) attached to sterile 27-gauge needles were used to harvest the CF which consists of the fluid fraction plus the cell suspension as the total immune cell population, through the soft peristomal membrane. CF was withdrawn in an equal ratio (1:1) with the sterile ice-cold anticoagulant solution CCM (Henson et al., 1999); or with a sterile ice-cold anticoagulant solution ISO-EDTA (Matranga et al., 2000); or with ASW only (Table 1). Selected media were chosen based on: (i) the normal chemical characteristics of CF in *P. lividus in vivo*, including pH and salinity; (ii) the results obtained from studies performed in the past using the same species and (iii) the known behavioural evidence concerning *P. lividus* immune cell growth and adherence to a surface. After their collection in these solutions, the immune cells were counted in a Fast-Read chamber (Biosigma), and morphological analysis of viable cells was performed using an optical microscope, Olympus CKX31 (Olympus, Japan). Cells harvested by selected media were compared with those collected without any type of solution. The Trypan Blue exclusion test was used to determine the number of viable cells present in the cell suspension. The immune cell suspension was kept on ice prior to seeding. All animals recovered after this procedure over a 15–20 day period.

**Table 1.**
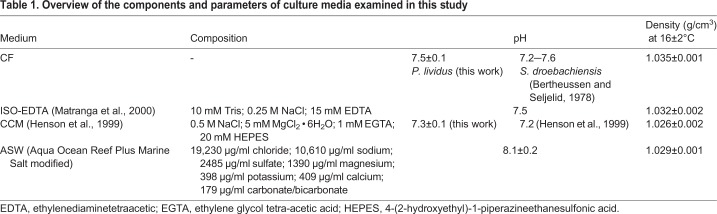
**Overview of the components and parameters of culture media examined in this study**

### Primary cell culture maintenance

The three different media were tested to determine which one was optimal for subsequent maintenance of cells in short-term and long-term primary cultures. All culturing experiments were performed by the deposition of the CF+CCM immune cell suspension on untreated plastic culture dishes, followed by the introduction of the selected autoclave-sterilised and 0.2 µm filtered medium (CCM, ISO-EDTA or ASW).

After immune cell counting, ≥1.5×10^6^ cells were seeded into each well of a 25-well plate (Thermo Fisher Scientific) and monitored for 48 h (short-term culture). For the long-term cultures, immune cells were plated at a density of ≥5×10^5^ cells/well and the procedure was carried out as follows: 6 h after the deposition of the CF+CCM immune cell suspension into the well, half of the medium was refreshed with sterile ASW supplemented with 0.12 mg/ml penicillin and 0.2 mg/ml streptomycin (P/S; Sigma-Aldrich). Subsequently, the entire volume of medium was replenished daily. Plates were kept at 16±2°C in the dark.

### Primary short-term cell culture validation based on a continuous-read viability measurement

All plate-based assays were done in a 96-well white, opaque-walled plate (Thermo Fisher Scientific) in a final volume of 100 µl. Immune cells were plated at a density of 1×10^5^ cells/well. Culturing was performed in the dark at 16±2°C. All assays involved at least five biological replicates (specimens). Continuous cell viability evaluation was performed by the RealTime-Glo MT Cell Viability Assay (Promega), according to the manufacturer's instructions, with minor changes as follows. At 12 h after seeding, 50 µl of the culture medium was replaced with fresh medium (CCM, ISO-EDTA or ASW) supplemented with the NanoLuc Luciferase Enzyme and the MT Cell Viability substrate (a cell-permeant pro-substrate) previously equilibrated to 16±2°C. Before the first measurement, reagents were incubated within the well for 30 min to obtain a stable signal. The luminescent/fluorescent signal was captured by the GloMax Discover System (Promega). The assay chemistry is based on the reducing potential of the cell, which is a known metabolic marker of cell viability. Briefly, viable cells reduce the cell-permeant pro-substrate to generate a substrate for NanoLuc Luciferase Enzyme. This substrate diffuses from the cells into the surrounding culture medium, where it is rapidly used by the NanoLuc Luciferase Enzyme to produce a luminescent signal captured by Glomax Discover System that is correlated to the number of viable cells. Cell cytotoxicity was determined by the non-lytic CellTox Green Cytotoxicity Assay (Promega). At 12 h post-seeding, 50 µl of the medium was replaced with fresh CCM, ISO-EDTA or ASW containing the CellTox Green Dye at a final dilution of 1 X. Immune cells were incubated for 30 min with CellTox Green Dye, prior to the first plate read (Ex: 485–500 nm; Em: 520–530 nm). The basis of this assay is the preferential binding of the cyanine dye to the DNA of dead cells, consequently activating the fluorescence of the dye in proportion to the level of cellular death.

### Phagocytic capacity assessment

To validate phagocytic function, we incubated the short-term primary culture with fluorescent nanobeads based on PD, Chromen 470-marked and carboxylated (Sigma-Aldrich), 5 µl of nanobeads per 1 ml of the culture medium (0.9×10^9^ nanobeads/ml). Incubation was performed in the dark for 1 h at 16±2°C. Cells were then fixed with methanol on ice for 5 min, and non-phagocytosed nanobeads were eliminated with two ASW wash steps. DNA dye Hoechst 33342 (Sigma-Aldrich), at a final concentration of 10 µg/ml, was added and incubated with the fixed primary culture for 7 min. Subsequently, cells were washed with three exchanges of ASW. A Zeiss Axioskop 2 Plus microscope (Zeiss, Arese, Italy), equipped for epifluorescence was used for image acquisition. Images were overlaid by using ImageJ software (NIH).

### Long-term primary cell culture evaluation with classical live staining dyes and morphological observation

The basic Trypan Blue exclusion test and Neutral Red (NR) assay were used in sequence to determine the number of viable cells and to monitor the lysosomal membrane stability of cultured cells for 2 weeks. An exclusion test was performed with 1 mg/ml Trypan Blue diluted in ASW at room temperature (RT) and cells (viable and non-viable) were immediately counted under the Zeiss Axioskop 2 Plus microscope or the Olympus CKX31 microscope (Olympus); images were recorded with a digital camera. Lysosomes of the cultured cells were labelled at RT in 4 µg/ml NR diluted in ASW for 1 h. Statistical analysis was carried out by counting ≥1000 cells in each of the three replicate wells. Assays were performed with samples from five to ten specimens.

### Statistical analysis

Statistical software GraphPad Prism Version 6.01 (USA) was used for the data processing. Statistical differences were estimated by the one-way analysis of variance (ANOVA) followed by Tukey's post-hoc test for multiple comparisons. The difference was deemed to be significant at *P*<0.05. Data were presented as the mean±standard deviation (s.d.).

## Supplementary Material

Supplementary information
