# Supplementary material for: Sea urchin Paracentrotus lividus immune cells in culture: formulation of the appropriate harvesting and culture media and maintenance conditions
Source: Biol Open. 2019 Feb 4;8(3):bio039289. doi: 10.1242/bio.039289 (PMC6451355; doi:10.1242/bio.039289)
Supplement: Supplementary information [file biolopen-8-039289-s1.pdf]

Supplementary Information

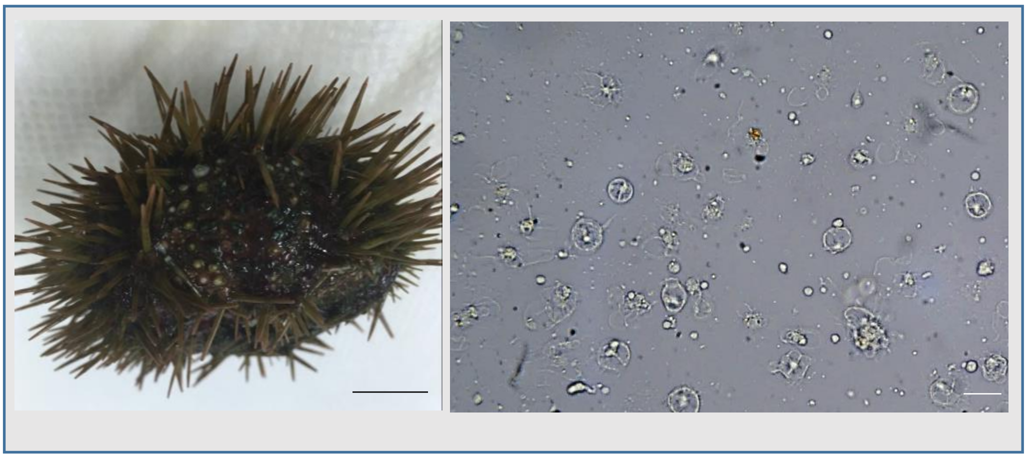

A B

Fig. S1. Host non-specific pathogens causing bald sea urchin disease induce collapse of the *in vitro–ex* vivo system. A) *P. lividus* affected by bald sea urchin disease showing spine loss and loss of the ectoderm (Bar, 1 cm); B) Frustrated phagocytic cells (after 24 h culture) are almost completely incapable of attaching to the culture dish and do not form cell–cell contacts. Amoebocytes and vibratile cells have undergone a severe degranulation event, leading to the almost complete disappearance of these immune cells. Bar, 10 µm.

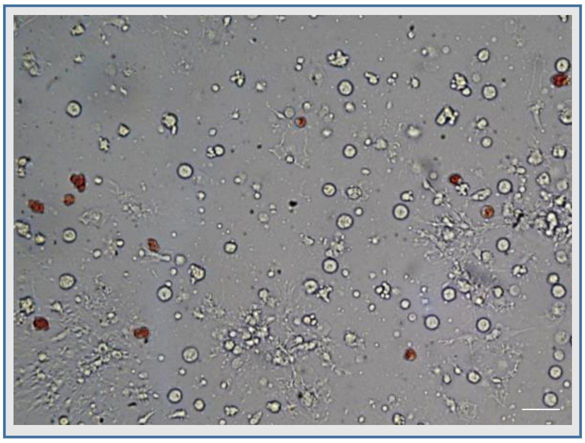

Fig. S2. Seeding an insufficient number of immune cells leads to culture failures. Specific signs of the failed culture shown in this picture include an absence of adhering cells, degranulation of amoebocytes and vibratile cell blockage. Bar, 10 µm.

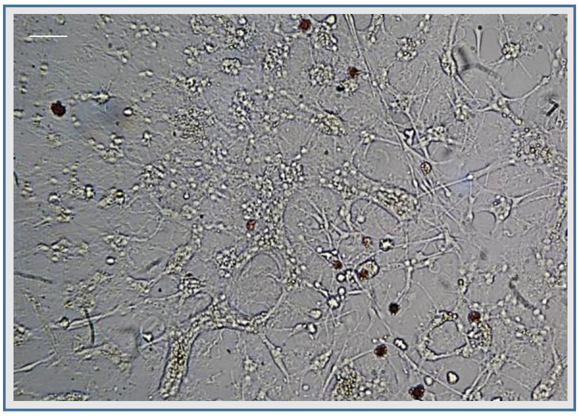

Fig. S3. Multinucleated syncitia observed after 48h in culture. When cells were cultured for 48 h without medium replacement, all nuclei formed bundles without clear nuclear or cytoplasmic borders.

Biology Open • Supplementary information

Table S1. Overview of the density parameters for selected harvesting and culturing media.

Medium	Salinity (ppt)	Density (g/cm^3)
**CF**	**46**	**1.035**
ASW	38	1.029
ISO-EDTA 2 X	84	1.063
CCM 2 X	66	1.051
CF + CCM 2 X	62	1.047
ISO-EDTA	42	1.032
(CF+CCM 2 X) + ISO-EDTA	55	1.042
CCM	34	1.026
**(CF + CCM 2 X) + CCM**	**44**	**1.033**
**(CF + CCM 2 X) + ASW**	**50**	**1.037**

CF, coelomic fluid; ASW, Artificial Seawater; ISO-EDTA, EDTA-containing buffer (Matranga et al., 2000); CCM, Coelomocyte Culture Medium (Henson et al., 1999); 2 X, 2 X Concentrated. Red text indicates culturing media presenting salinity and density very close that of the CF.

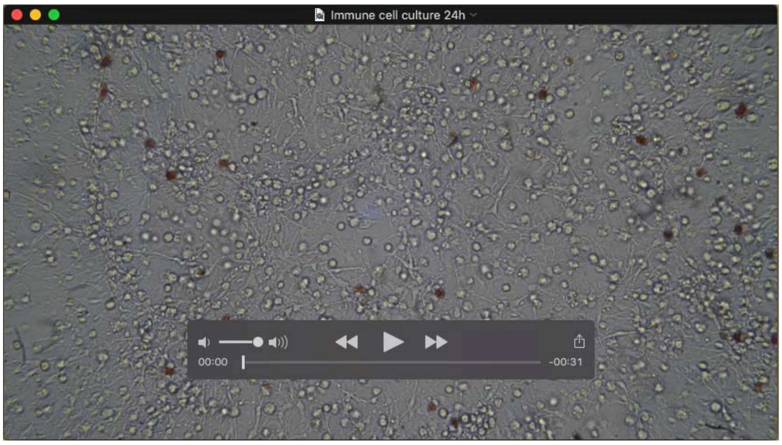

Movie 1. ***P. lividus*** **primary immune cell culture after 24 h in culture in CCM.**
